# Supplementary material for: Portland and Belite Cement Hydration Acceleration by C-S-H Seeds with Variable w/c Ratios
Source: Materials (Basel). 2022 May 16;15(10):3553. doi: 10.3390/ma15103553 (PMC9147420; doi:10.3390/ma15103553)
Supplement: Supplementary file 1 [file materials-15-03553-s001.zip › materials-1723666-supplementary.pdf]

materials

Supporting Information

Portland and Belite cement hydration acceleration by C-S-H seeds with variable w/c ratios

Alejandro Morales-Cantero[1], Ana Cuesta[1], Angeles G. De la Torre[1], Oliver Mazanec[2], Pere Borralleras[3], Kai Steffen Weldert[2], Daniela Gastaldi[4], Fulvio Canonico[4], Miguel A. G. Aranda[1*]

[1] Departamento de Química Inorgánica, Cristalografía y Mineralogía, Universidad de Málaga, Málaga, 29071, Spain
[2] *Master Builders Solutions Deutschland GmbH, Albert-Frank Str. 32, 83308 Trostberg, Germany*
[3] *Master Builders Solutions España S.L.U., Carretera de l'Hospitalet, 147-149, Edificio Viena, 1ª planta, 08940 Cornellà de Llobregat, Spain*
[4] *Research & Development, Buzzi Unicem, Via Luigi Buzzi 6, 15033 Casale Monferrato, Italy*
* *email: g_aranda@uma.es*

This file contains

→ Nine tables:

Table S1. Elemental composition (wt%) from XRF for the three anhydrous cements.
Table S2. Mineralogical composition (wt%) by Rietveld quantitative phase analysis from LXRPD, MoKα1 (λ=0.7093 Å), for the three anhydrous cements.
Table S3. Textural analysis for the three anhydrous cements.
Table S4. RQPA results for PC-42.5-wc05 pastes.
Table S5. RQPA results for PC-42.5-wc04 pastes.
Table S6. RQPA results for BC-Buz-wc05 pastes.
Table S7. RQPA results for BC-Buz-wc04 pastes.
Table S8. RQPA results for BC-n.a.-wc05 pastes.
Table S9. RQPA results for BC-n.a.-wc04 pastes.

→ Detailed additional information:

♦ Theoretical calculations for (i) the free water content determination, (ii) the C-S-H gel content, and (iii) the remaining amorphous, other-ACn, content. All these calculations are based on the RQPA results by assuming some stoichiometries for the chemical reactions.

♦ Raw data availability.

→ Three figures:

Figure S1. Laboratory X-ray powder diffraction, MoKα1 (λ=0.7093 Å), for pastes with w/c=0.40 at 28d: (a) PC-42.5-wc04-Ref and (b) PC-42.5-wc04-STE53.
Figure S2. Laboratory X-ray powder diffraction, MoKα1 (λ=0.7093 Å), for pastes with w/c=0.40 at 28d: (a) BC-Buz-wc04-Ref and (b) BC-Buz-wc04-STE53.
Figure S3. Laboratory X-ray powder diffraction, MoKα1 (λ=0.7093 Å), for pastes with w/c=0.40 at 28d: (a) BC-n.a.-wc04-Ref and (b) BC-n.a.-wc04-STE53.

Table S1. Elemental composition (wt%) from XRF for the three anhydrous cements.

	CaO	SiO_2	SO_3	Al_2O_3	Fe_2O_3	MgO	K_2O	Na_2O	Others	LoI
**PC-42.5**	62.9	19.7	3.4	5.0	3.4	1.5	1.1	0.3	0.3	2.7
**BC-Buz**	59.3	21.4	4.8	4.3	2.8	2.7	0.9	0.2	0.9	2.9
**BC-n.a.**	60.1	24.4	2.8	2.9	4.8	1.2	0.5	0.1	1.3	2.0

Table S2. Mineralogical composition (wt%) by Rietveld quantitative phase analysis from LXRPD, MoKα1 (λ=0.7093 Å), for the three anhydrous cements.

	C_3S*	β-C_2S	C_4AF	C_3A#	$C_4A_3\bar{S}$	MgO	$C\bar{C}$	$CM\bar{C}_2$	$C\bar{S}H_2$	$C\bar{S}H_{0.5}$	$C\bar{S}$	ACn
**PC-42.5**	54.2	9.3	9.8	6.8	-	-	4.2	0.7	1.0	1.1	-	13.0
**BC-Buz**	24.8	44.1	10.1	2.0	1.9	1.4	1.3	-	-	-	1.6	12.8
**BC-n.a.**	28.6	48.0	15.2	-	-	-	-	-	2.0	-	-	6.2

*PC-42.5: C_3S-M3; BC-Buz: 2.9 wt% C_3S-M3 and 21.9 wt% C_3S-M1; BC-n.a.: 1.8 wt% C_3S-M3 and 26.8 wt% C_3S-M1
#PC-42.5: o-C_3A; BC-Buz: c-C_3A

Table S3. Textural analysis for the three anhydrous cements.

	Density (g/cm³)	BET (m²/g)	Blaine (m²/Kg)	$D_{v,10}$ (μm)	$D_{v,50}$ (μm)	$D_{v,90}$ (μm)
**PC-42.5**	3.13	1.2	370	3.6	18.1	50.3
**BC-Buz**	3.17	1.4	502	1.5	12.8	55.2
**BC-n.a.**	3.23	1.5	466	1.1	7.1	38.4

Table S4. RQPA results for PC-42.5-wc05 pastes. The cement also contained at t_0 0.7 wt% of $C\bar{S}H_2$ and 0.7 wt% of $C\bar{S}H_{0.5}$.

**Phases**	**t_0**	**PC-42.5-wc05-Ref**			**PC-42.5-wc05-XS130**			**PC-42.5-wc05-STE53**		
		**1d**	**7d**	**28d**	**1d**	**7d**	**28d**	**1d**	**7d**	**28d**
C_3S	36.1	16.7	8.1	3.8	16.4	8.4	4.2	14.7	8.7	2.7
C_2S	6.2	6.1	5.4	5.8	6.2	6.0	5.7	6.5	7.0	5.8
C_4AF	6.6	6.1	4.6	3.3	5.4	3.6	2.1	5.4	3.8	2.3
C_3A	4.5	4.2	2.0	0.5	3.5	1.8	0.6	3.3	1.6	0.5
$C\bar{C}$	2.8	4.9	5.2	3.1	6.6	4.3	2.4	5.9	2.4	2.2
$CM\bar{C}_2$	0.5	0.4	0.7	0.9	0.4	0.7	0.7	0.4	0.8	0.6
CH	-	6.5	10.2	12.3	5.7	8.9	11.1	7.1	10.0	10.8
AFt	-	5.0	8.4	8.0	6.1	9.0	8.5	5.8	7.5	7.0
AFm-Hc	-	-	-	-	-	1.1	0.9	-	0.7	0.7
AFm-Mc	-	-	-	1.2	-	-	1.1	-	-	1.8
ACn	8.7	24.4	33.8	42.9	25.5	36.8	45.4	25.4	37.8	49.9
FW_{TA}#	33.3	25.5	21.4	18.1	24.0	19.4	17.3	25.3	19.8	15.8
C-S-H$	-	*19.9*	*29.6*	*33.5*	*20.1*	*28.5*	*33.2*	*21.4*	*26.9*	*34.6*
Other ACn$	*8.7*	*5.4*	*9.2*	*15.9*	*5.9*	*11.7*	*18.1*	*7.2*	*14.5*	*20.4*
FW$	*33.3*	*24.6*	*16.3*	*11.6*	*23.5*	*15.9*	*11.4*	*22.1*	*16.2*	*10.7*

FW (free water) content determined from thermal data; $ In italics, calculated data of the chemical reaction

Table S5. RQPA results for PC-42.5-wc04 pastes. The cement also contained at t_0 0.7 wt% of $C\bar{S}H_2$ and 0.8 wt% of $C\bar{S}H_{0.5}$.

**Phases**	**t_0**	**PC-42.5-wc04-Ref**			**PC-42.5-wc04-XS130**			**PC-42.5-wc04-STE53**		
		**1d**	**7d**	**28d**	**1d**	**7d**	**28d**	**1d**	**7d**	**28d**
C_3S	38.7	18.2	8.6	4.5	17.6	9.8	6.7	16.8	9.5	4.9
C_2S	6.6	6.9	6.7	7.2	7.0	7.1	7.1	7.1	7.8	7.5
C_4AF	7.0	6.4	5.0	4.3	5.6	3.9	3.5	5.9	3.8	2.6
C_3A	4.8	4.5	2.0	0.8	3.6	1.4	0.8	3.7	1.2	0.3
$C\bar{C}$	3.0	5.4	5.7	4.5	5.7	3.6	3.1	5.6	3.6	2.3
$CM\bar{C}_2$	0.5	0.5	0.3	0.4	0.6	0.6	0.5	0.6	0.8	0.9
CH	-	6.7	9.9	11.3	6.2	7.9	9.5	7.2	9.2	10.3
AFt	-	5.1	8.2	8.2	6.2	7.0	6.3	5.5	6.3	5.8
AFm-Hc	-	-	-	0.3	-	0.7	0.3	-	1.0	0.4
AFm-Mc	-	-	-	1.2	-	1.3	1.5	-	-	2.0
ACn	9.3	26.4	38.9	45.1	28.5	42.0	49.4	28.7	42.8	52.9
FW_{TA}#	28.6	*19.7*	*14.8*	*12.3*	*19.1*	*14.7*	*11.4*	*18.8*	*14.0*	*10.2*
C-S-H$	-	*20.9*	*30.7*	*34.9*	*21.5*	*29.5*	*32.6*	*22.3*	*29.8*	*34.5*
Other ACn$	*9.3*	*5.8*	*12.4*	*16.0*	*8.8*	*17.7*	*21.1*	*7.9*	*18.0*	*23.6*
FW$	*28.6*	*19.4*	*10.6*	*6.5*	*17.3*	*9.5*	*7.1*	*17.2*	*9.0*	*5.1*

FW (free water) content determined from thermal data; $ In italics, calculated data of the chemical reaction

Table S6. RQPA results for BC-Buz-wc05 pastes. The cement also contained at t_0 1.0 wt% of $C\overline{S}$.

**Phases**	t_0	**BC-Buz-wc05-Ref**			**BC-Buz-wc05-XS130**			**BC-Buz-wc05-STE53**		
		**1d**	**7d**	**28d**	**1d**	**7d**	**28d**	**1d**	**7d**	**28d**
C_3S	16.6	5.4	4.2	3.6	5.3	4.2	2.8	5.0	3.4	2.6
C_2S	29.4	33.5	28.0	18.7	32.0	23.8	16.0	31.8	27.7	18.1
C_4AF	6.7	5.5	4.0	1.7	3.3	1.7	1.1	3.1	1.6	0.9
C_3A	1.3	0.4	-	-	-	-	-	-	-	-
$C_4A_3\overline{S}$	1.2	0.7	-	-	-	-	-	0.3	-	-
$C\overline{C}$	0.9	3.1	0.9	1.2	2.6	1.3	0.9	2.7	0.9	0.5
MgO	0.9	1.1	0.9	0.8	1.0	0.9	0.9	1.0	0.9	0.7
CH	-	2.1	3.5	4.0	1.5	2.9	3.5	1.7	3.1	3.7
AFt	-	7.1	8.9	13.1	9.6	12.9	12.5	9.4	9.0	11.4
AFm-Hc	-	-	0.5	0.7	1.4	2.4	1.7	1.0	0.9	1.7
AFm-Mc	-	-	-	1.2	-	-	-	-	1.9	2.3
Katoite	-	-	0.5	0.8	-	0.5	0.5	-	0.5	0.5
ACn	8.6	15.1	26.5	34.1	18.5	28.5	42.1	19.1	29.2	40.4
FW_{TA}#	33.3	26.0	22.1	20.1	24.9	21.0	18.0	25.0	21.1	17.1
C-S-H$	-	*11.4*	*14.5*	*27.7*	*11.5*	*20.2*	*32.2*	*11.8*	*15.8*	*29.5*
Other ACn$	*8.6*	*3.6*	*11.2*	*9.0*	*8.3*	*9.3*	*12.2*	*8.5*	*13.2*	*11.5*
FW$	*33.3*	*26.1*	*22.8*	*17.5*	*23.6*	*20.0*	*15.8*	*23.7*	*21.4*	*16.5*

FW (free water) content determined from thermal data; $ In italics, calculated data of the chemical reaction

Table S7. RQPA results for BC-Buz-wc04 pastes. The cement also contained at t_0 1.1 wt% of $C\overline{S}$.

**Phases**	t_0	**BC-Buz-wc04-Ref**			**BC-Buz-wc04-XS130**			**BC-Buz-wc04-STE53**		
		**1d**	**7d**	**28d**	**1d**	**7d**	**28d**	**1d**	**7d**	**28d**
C_3S	17.7	6.1	3.7	2.7	6.3	3.4	3.0	5.9	3.4	3.2
C_2S	31.5	35.9	28.9	19.9	35.9	30.4	21.2	36.0	34.7	21.1
C_4AF	7.2	6.1	3.5	1.9	2.9	2.1	1.5	2.9	1.8	0.8
C_3A	1.4	0.6	-	-	0.3	-	-	-	-	-
$C_4A_3\overline{S}$	1.3	0.8	-	-	-	-	-	0.3	-	-
$C\overline{C}$	0.9	3.3	5.7	3.8	2.4	0.6	0.3	2.4	0.3	-
MgO	1.0	1.1	0.9	0.9	1.2	1.2	1.0	1.2	1.2	1.0
CH	-	2.0	3.3	4.2	1.0	1.5	2.7	1.5	1.6	3.2
AFt	-	6.8	10.0	11.0	9.2	12.3	8.9	8.2	11.2	8.8
AFm-Hc	-	-	-	0.2	1.3	2.8	1.2	1.1	2.5	1.3
AFm-Mc	-	-	1.1	2.1	-	-	-	-	-	-
Katoite	-	-	0.5	0.7	-	0.5	0.7	-	0.4	0.5
ACn	9.2	15.7	24.7	38.0	19.8	29.8	47.3	21.5	26.5	47.9
FW_{TA}#	28.6	21.5	17.8	14.6	19.7	15.5	12.2	19.0	16.4	12.2
C-S-H$	-	*11.8*	*17.8*	*31.0*	*11.6*	*16.1*	*28.9*	*12.0*	*14.6*	*28.8*
Other ACn$	*9.2*	*3.9*	*8.5*	*10.4*	*9.2*	*13.0*	*18.9*	*10.1*	*11.8*	*19.8*
FW$	*28.6*	*21.4*	*16.2*	*11.2*	*18.7*	*16.2*	*11.7*	*18.4*	*16.5*	*11.5*

FW (free water) content determined from thermal data; $ In italics, calculated data of the chemical reaction

Table S8. RQPA results for BC-n.a.-wc05 pastes.

Phases	t_0	BC-n.a.-wc05-Ref			BC-n.a.-wc05-XS130			BC-n.a.-wc05-STE53		
		**1d**	**7d**	**28d**	**1d**	**7d**	**28d**	**1d**	**7d**	**28d**
C_3S	19.0	6.9	2.4	1.7	8.1	2.3	1.2	6.9	3.0	1.3
C_2S	32.1	33.1	34.5	24.9	34.2	33.7	17.9	31.8	34.4	21.9
C_4AF	10.1	9.0	6.4	6.7	6.5	5.3	2.9	7.7	5.9	3.1
$C\bar{S}H_2$	1.4	0.4	0.3	0.4	0.4	0.3	0.5	0.5	0.5	0.6
$C\bar{C}$	-	2.2	-	-	1.8	-	-	1.2	-	-
CH	-	3.0	5.0	4.9	2.2	4.2	5.1	3.1	4.9	4.7
AFt	-	4.0	5.2	3.0	4.0	4.8	4.3	4.5	4.9	3.9
AFm-Hc	-	-	0.3	0.4	-	0.4	0.6	-	0.3	0.7
ACn	4.1	14.0	21.6	37.6	16.2	24.9	47.6	17.3	21.6	43.6
FW_{TA} #	33.3	27.5	24.3	20.4	26.6	24.1	19.9	27.0	24.4	20.2
C-S-H $	-	*12.3*	*16.9*	*27.4*	*11.1*	*17.0*	*37.3*	*12.3*	*16.3*	*31.8*
Other ACn $	*4.1*	*1.3*	*4.1*	*9.0*	*4.4*	*7.7*	*13.5*	*4.7*	*4.7*	*13.4*
FW $	*33.3*	*27.9*	*24.9*	*21.6*	*27.3*	*24.4*	*16.7*	*27.3*	*24.9*	*18.6*

FW (free water) content determined from thermal data; $ In italics, calculated data of the chemical reaction

Table S9. RQPA results for BC-n.a.-wc04 pastes.

Phases	t_0	BC-n.a.-wc04-Ref			BC-n.a.-wc04-XS130			BC-n.a.-wc04-STE53		
		**1d**	**7d**	**28d**	**1d**	**7d**	**28d**	**1d**	**7d**	**28d**
C_3S	20.4	8.5	3.6	1.5	10.4	2.9	3.1	7.5	2.8	2.5
C_2S	34.2	35.9	35.4	27.9	35.5	35.4	20.6	35.4	37.2	24.9
C_4AF	10.9	9.2	7.3	6.7	8.2	5.6	2.9	8.6	6.0	2.6
$C\bar{S}H_2$	1.5	0.5	0.7	0.5	0.6	0.6	0.6	0.4	0.5	0.7
$C\bar{C}$	-	1.8	-	-	0.6	-	-	0.4	-	-
CH	-	3.9	4.4	4.5	2.1	5.3	5.0	3.2	5.5	6.5
AFt	-	3.8	5.0	3.8	5.1	5.0	3.6	5.7	4.9	3.5
AFm-Hc	-	-	0.4	0.6	-	-	0.4	-	-	0.6
ACn	4.4	14.0	23.8	39.3	15.2	26.2	50.5	17.3	23.9	44.7
FW_{TA} #	28.6	22.5	19.4	15.2	22.4	19.0	13.3	21.6	19.2	14.1
C-S-H $	-	*12.1*	*17.1*	*27.8*	*10.2*	*17.9*	*36.0*	*13.2*	*18.0*	*30.8*
Other ACn $	*4.4*	*1.4*	*5.9*	*10.4*	*4.1*	*8.2*	*15.7*	*2.4*	*5.9*	*14.4*
FW $	*28.6*	*23.0*	*20.2*	*16.3*	*23.3*	*19.1*	*12.1*	*23.3*	*19.3*	*13.6*

FW (free water) content determined from thermal data; $ In italics, calculated data of the chemical reaction

♦ **Theoretical calculations of the free water, the C-S-H gel, and the other ACn contents based on chemical reactions and the RQPA results.**

For the theoretical calculation of free water, equations (1) to (5) were used; equations (1) and (2) were been used for the calculation of C-S-H gel

$$Ca_3SiO_5 + 5.2H_2O \rightarrow 1.2Ca(OH)_2 + (CaO)_{1.8}SiO_2(H_2O)_{4.0} \quad (1)$$

$$Ca_2SiO_5 + 4.2H_2O \rightarrow 0.2Ca(OH)_2 + (CaO)_{1.8}SiO_2(H_2O)_{4.0} \quad (2)$$

$$Ca_3Al_2O_6 + 3CaSO_4 + 32H_2O \rightarrow Ca_6Al_2(SO_4)_3(OH)_{12}{\cdot}26H_2O \quad (3)$$

$$Ca_4Al_6O_{12}(SO_4) + 2CaSO_4 + 38H_2O \rightarrow Ca_6Al_2(OH)_{12}(SO_4)_3{\cdot}26H_2O + 4Al(OH)_3 \quad (4)$$

$$Ca_4Al_2Fe_2O_{10} + 3CaSO_4{\cdot}2H_2O + 30H_2O \rightarrow Ca_6Al_2(OH)_{12}(SO_4)_3{\cdot}26H_2O + Ca(OH)_2 + 2Fe(OH)_3 \quad (5)$$

Detailed example for free water (FW) calculation. The FW at a given hydration time was calculated for each sample and age, using the initial free water and considering the bounded water according to the stoichiometry of reactions (1) to (5).

The calculations for BC-Buz-wc04-Ref at 28d are detailed next as an example; see Table S7.

I. C_3S: There is 2.7 wt% of unreacted C_3S. This means that 15.0 wt% ($C_3S_{initial} – C_3S_{final}$) has reacted. According to reaction (1), 6.1 wt% of free water has been consumed.

II. C_2S: There is 19.9 wt% of unreacted C_2S. This means that 11.6 wt% ($C_2S_{initial} – C_2S_{final}$) has reacted. According to reaction (2), 5.1 wt% of free water has been consumed.

III. C_3A: All C_3A has been dissolved at this age. This means that 1.4 wt% ($C_3A_{initial}$ – C_3A_{final}) has reacted. According to reaction (3), 2.4 wt% of free water has been consumed.

IV. $C_4A_3\bar{S}$: All $C_4A_3\bar{S}$ has been dissolved at this age. This means that 1.3 wt% ($C_4A_3\bar{S}_{initial}$ – $C_4A_3\bar{S}_{final}$) has reacted. According to reaction (4), 1.5 wt% of free water has been consumed.

V. C_4AF: There is 1.9 wt% of unreacted C_4AF. This means that 5.3 wt% ($C_4AF_{initial}$ – C_4AF_{final}) has reacted. According to reaction (5), 2.3 wt% of free water has been consumed.

The calculated free water content for BC-Buz-wc04-Ref at 28d is **11.2 wt%** (28.6 wt% - 6.1 wt% - 5.1 wt% - 2.4 wt% - 1.5 wt% - 2.3 wt%)

Detailed example for C-S-H gel calculation. C-S-H gel was calculated according to reactions (1) and (2). The calculations for BC-Buz-wc04-Ref at 28d are detailed as an example; see Table S7.

I. C_3S: There is 2.7 wt% of unreacted C_3S. This means that 15.0 wt% ($C_3S_{initial} – C_3S_{final}$) has reacted. According to reaction (1), 15.3 wt% of C-S-H gel has been formed.

II. C_2S: There is 19.9 wt% of unreacted C_2S. This means that 11.6 wt% ($C_2S_{initial} – C_2S_{final}$) has reacted. According to reaction (2), 15.7 wt% of C-S-H gel has been formed.

A total of **31.0 wt%** of C-S-H gel has been formed.

Detailed example for other-ACn calculation. The calculated FW and C-S-H gel should be close to the total ACn content. Differences between total ACn and calculated C-S-H and FW are considered to be other-ACn (such as AFm-type, iron-siliceous hydrogarnet, and the remaining fraction of amorphous phase(s) within the unreacted clinker components).

BC-Buz-wc04-Ref at 28d is used as an example; see Table S7.

The difference of total ACn obtained by internal standard (52.6 wt%) and free water (11.2 wt%) and the calculated C-S-H gel (31.0 wt%) give the other-ACn contents (52.6 wt% - 11.2 wt% - 31.0 wt% = **10.4 wt%** other-ACn)

♦ **Raw data availability.**

Raw data from the following techniques have been deposited: Calorimetry (excel file), LXRPD (txt file) MIP (excel file), TA (TA Universal Analysis software file). Data can be accessed at: https://doi.org/10.5281/zenodo.6335735

Calorimetry

PC-42.5-wc05-Ref (1 file)
PC-42.5-wc05-XS130 (1 file)
PC-42.5-wc05-STE53 (1 file)
PC-42.5-wc04-Ref (1 file)
PC-42.5-wc04-XS130 (1 file)
PC-42.5-wc04-STE53 (1 file)
BC-Buz-wc05-Ref (1 file)
BC-Buz-wc05-XS130 (1 file)
BC-Buz-wc05-STE53 (1 file)
BC-Buz-wc04-Ref (1 file)
BC-Buz-wc04-XS130 (1 file)
BC-Buz-wc04-STE53 (1 file)
BC-n.a.-wc05-Ref (1 file)
BC-n.a.-wc05-XS130 (1 file)
BC-n.a.-wc05-STE53 (1 file)
BC-n.a.-wc04-Ref (1 file)
BC-n.a.-wc04-XS130 (1 file)
BC-n.a.-wc04-STE53 (1 file)

MIP

PC-42.5-wc05-Ref-Serie (3 files)
PC-42.5-wc05-XS130-Serie (3 files)
PC-42.5-wc05-STE53-Serie (3 files)
PC-42.5-wc04-Ref-Serie (3 files)
PC-42.5-wc04-XS130-Serie (3 files)
PC-42.5-wc04-STE53-Serie (3 files)
BC-Buz-wc05-Ref-Serie (3 files)
BC-Buz-wc05-XS130-Serie (3 files)
BC-Buz-wc05-STE53-Serie (3 files)
BC-Buz-wc04-Ref-Serie (3 files)
BC-Buz-wc04-XS130-Serie (3 files)
BC-Buz-wc04-STE53-Serie (3 files)
BC-n.a.-wc05-Ref-Serie (3 files)
BC-n.a.-wc05-XS130-Serie (3 files)
BC-n.a.-wc05-STE53-Serie (3 files)
BC-n.a.-wc04-Ref-Serie (3 files)
BC-n.a.-wc04-XS130-Serie (3 files)
BC-n.a.-wc04-STE53-Serie (3 files)

LXRPD

PC-42.5-Anh (1 file)
PC-42.5-wc05-Ref-Serie (3 files)
PC-42.5-wc05-XS130-Serie (3 files)
PC-42.5-wc05-STE53-Serie (3 files)
PC-42.5-wc04-Ref-Serie (3 files)
PC-42.5-wc04-XS130-Serie (3 files)
PC-42.5-wc04-STE53-Serie (3 files)
BC-Buz-Anh (1 file)
BC-Buz-wc05-Ref-Serie (3 files)
BC-Buz-wc05-XS130-Serie (3 files)
BC-Buz-wc05-STE53-Serie (3 files)
BC-Buz-wc04-Ref-Serie (3 files)
BC-Buz-wc04-XS130-Serie (3 files)
BC-Buz-wc04-STE53-Serie (3 files)
BC-n.a.-Anh (1 file)
BC-n.a.-wc05-Ref-Serie (3 files)
BC-n.a.-wc05-XS130-Serie (3 files)
BC-n.a.-wc05-STE53-Serie (3 files)
BC-n.a.-wc04-Ref-Serie (3 files)
BC-n.a.-wc04-XS130-Serie (3 files)
BC-n.a.-wc04-STE53-Serie (3 files)

TA

PC-42.5-wc05-Ref-Serie (3 files)
PC-42.5-wc05-XS130-Serie (3 files)
PC-42.5-wc05-STE53-Serie (3 files)
PC-42.5-wc04-Ref-Serie (3 files)
PC-42.5-wc04-XS130-Serie (3 files)
PC-42.5-wc04-STE53-Serie (3 files)
BC-Buz-wc05-Ref-Serie (3 files)
BC-Buz-wc05-XS130-Serie (3 files)
BC-Buz-wc05-STE53-Serie (3 files)
BC-Buz-wc04-Ref-Serie (3 files)
BC-Buz-wc04-XS130-Serie (3 files)
BC-Buz-wc04-STE53-Serie (3 files)
BC-n.a.-wc05-Ref-Serie (3 files)
BC-n.a.-wc05-XS130-Serie (3 files)
BC-n.a.-wc05-STE53-Serie (3 files)
BC-n.a.-wc04-Ref-Serie (3 files)
BC-n.a.-wc04-XS130-Serie (3 files)
BC-n.a.-wc04-STE53-Serie (3 files)

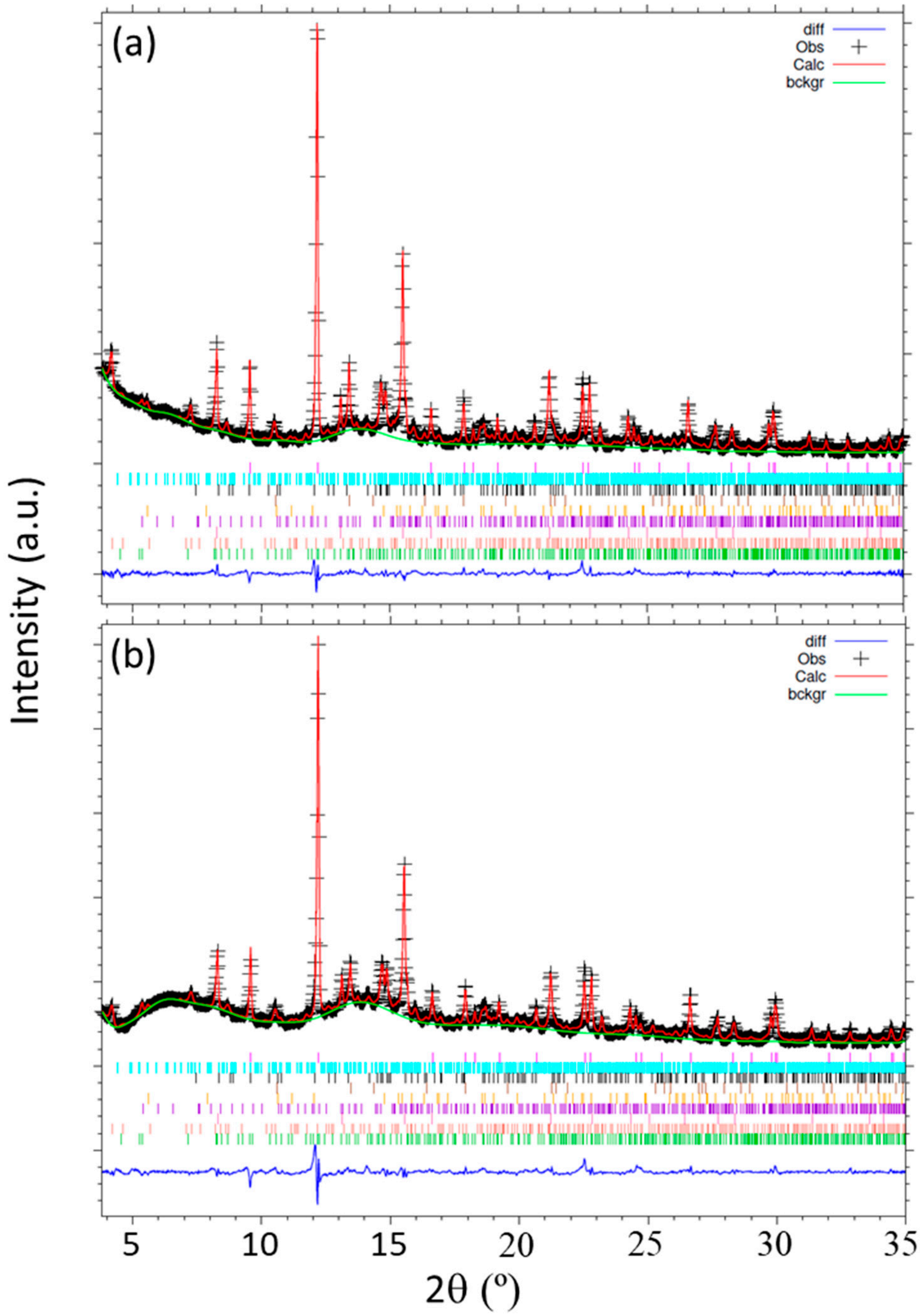

Figure S1. Laboratory X-ray powder diffraction, MoKα_1 (λ=0.7093 Å), for pastes with w/c=0.40 at 28d: (a) PC-42.5-wc04-Ref and (b) PC-42.5-wc04-STE53.

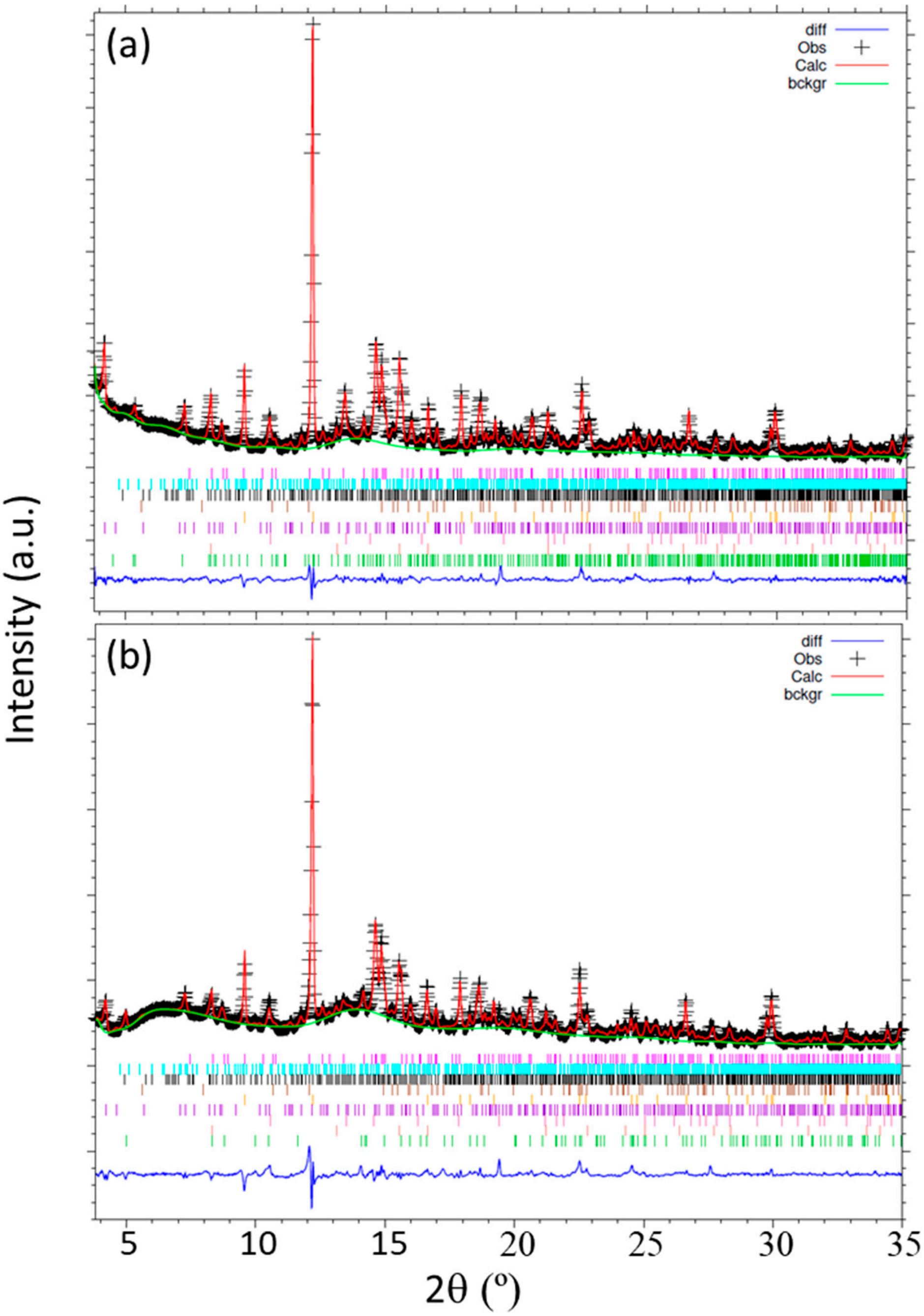

Figure S2. Laboratory X-ray powder diffraction, MoKα_1 (λ=0.7093 Å), for pastes with w/c=0.40 at 28d: (a) BC-Buz-wc04-Ref and (b) BC-Buz-wc04-STE53.

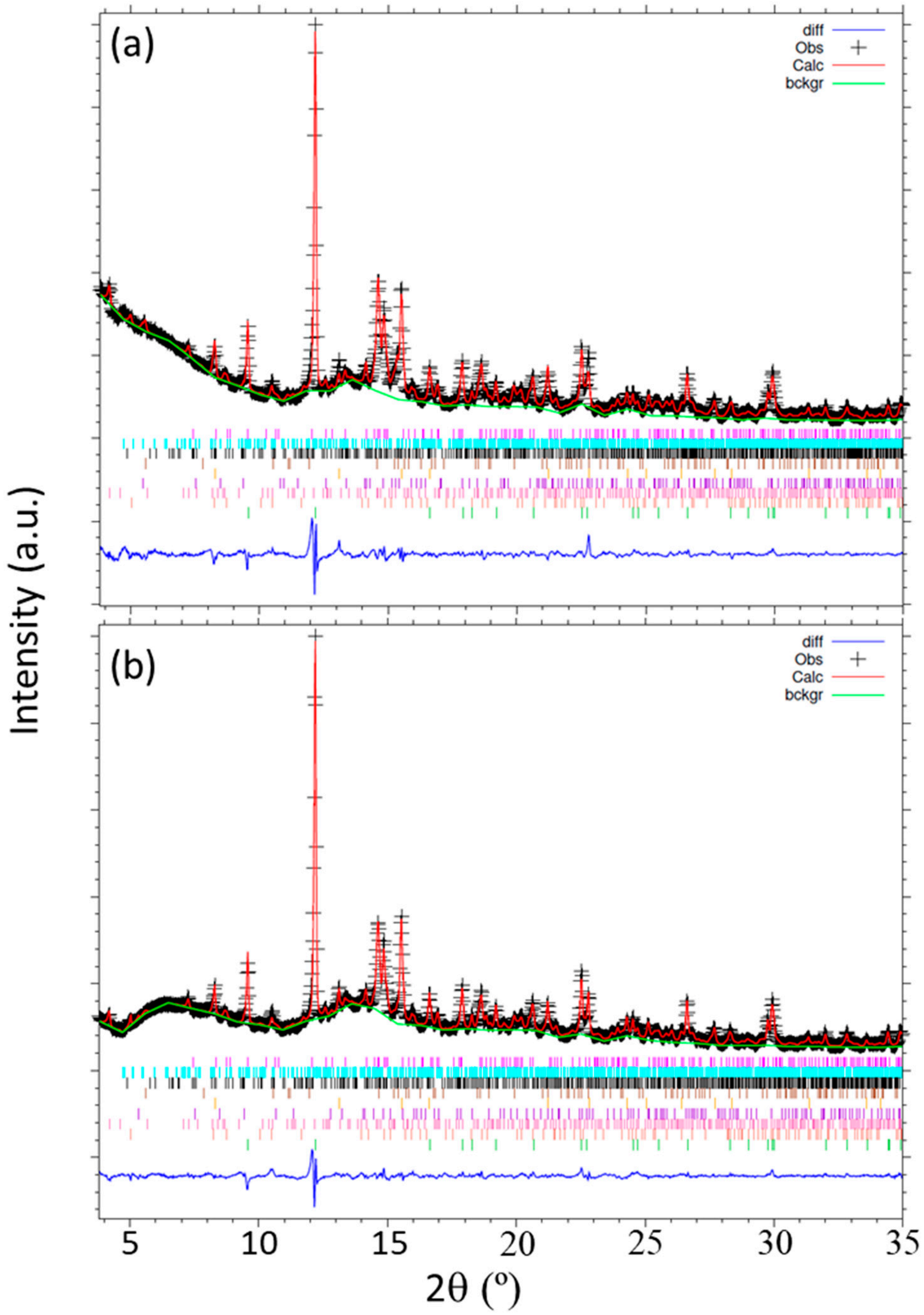

Figure S3. Laboratory X-ray powder diffraction, MoKα_1 (λ=0.7093 Å), for pastes with w/c=0.40 at 28d: (a) BC-n.a.-wc04-Ref and (b) BC-n.a.-wc04-STE53.
